# Autophagy- An emerging target for melanoma therapy

**DOI:** 10.12688/f1000research.8347.1

**Published:** 2016-07-29

**Authors:** Abibatou Ndoye, Ashani T. Weeraratna

**Affiliations:** 1 Melanoma Research Center, Wistar Institute, Philadelphia, PA, USA; 2 University of the Sciences in Philadelphia, Philadelphia, PA, USA

**Keywords:** autophagy, tumor, melanoma

## Abstract

Melanoma accounts for only 5% of all cancers but is the leading cause of skin cancer death due to its high metastatic potential. Patients with metastatic melanoma have a 10-year survival rate of less than 10%. While the clinical landscape for melanoma is evolving rapidly, lack of response to therapies, as well as resistance to therapy remain critical obstacles for treatment of this disease. In recent years, a myriad of therapy resistance mechanisms have been unravelled, one of which is autophagy, the focus of this review. In advanced stages of malignancy, melanoma cells hijack the autophagy machinery in order to alleviate drug-induced and metabolic stress in the tumor microenvironment, thereby promoting resistance to multiple therapies, tumor cell survival, and progression.  Autophagy is an essential cellular process that maintains cellular homeostasis through the recycling of intracellular constituents. Early studies on the role of autophagy in cancer generated controversy as to whether autophagy was pro- or anti-tumorigenic. Currently, there is a consensus that autophagy is tumor-suppressive in the early stages of cancer and tumor-promoting in established tumors.  This review aims to highlight current understandings on the role of autophagy in melanoma malignancy, and specifically therapy resistance; as well as to evaluate recent strategies for therapeutic autophagy modulation.

## Introduction

Malignant melanoma arises from melanocytes, cells that are responsible for pigment production. Approximately 50% of melanomas harbor a mutation in the BRAF gene, which leads to the dysregulated downstream activation of the MEK/ERK signaling pathway ^1^. BRAF is a serine/threonine kinase that is involved in various cellular processes such as proliferation, differentiation, and survival ^2^. An important approach to treatment for melanoma is the use of inhibitors such as vemurafenib, dabrafenib, or cobimetinib, which specifically target the mutant BRAF. BRAF-directed therapy initially decreases tumor burden considerably; however, most patients ultimately develop resistance and tumors recur ^3– 5^. Melanoma cells can adopt various mechanisms of resistance such as the re-activation of the mitogen-activated protein kinase (MAPK) signaling pathway ^6^ and aberrant activation of other signaling pathways such as the insulin-like growth factor receptor (IGFR) and Wnt signaling pathways ^7, 8^. Ultimately, reactivation of the MAPK pathway is a key feature of all of these different resistance mechanisms, and this has prompted the approval of combinatorial MAPK therapies that target multiple nodes in this signaling pathway ^9^. In addition to these approaches to inhibit MAPK signaling for therapeutic gain, another important form of therapy for melanoma is immune checkpoint inhibition. This emerging form of therapy targets molecules such as CTLA-4 (cytotoxic T-lymphocyte-associated protein 4) or PD-1 (programmed cell death protein 1), which are found on immune cells and signal to inhibit the activity of T cells ^10^. By disabling these checkpoints, most often by using antibodies against CTLA-4 or PD-1, the immune system is re-activated and can act to target and destroy the tumor cells. When these therapies are effective, their effects are durable, but many still do not initially respond to treatment, and resistance still appears in a subset of those who do respond, with the most recent data suggesting that while these rates are slightly increased with combination approaches to immunotherapy, they still remain under 50%. Understanding the mechanisms of resistance to targeted therapy and immunotherapy is therefore critical for effective eradication of melanoma.

The signaling pathways involved in resistance as well as the spectrum of therapy-acquired mutations that may arise have been well studied and discussed, while the cellular aspects of therapy resistance have not. For example, one way for melanoma cells to resist therapy is to undergo an adaptive stress response. We have previously shown that the initiation of a senescent-like phenotype is one way in which melanoma cells resist vemurafenib ^11^. Recent studies in various types of cancers such as colorectal cancer, prostate cancer, lung cancer, and melanoma have demonstrated that in established tumors, cancer cells can also upregulate a related stress response process: autophagy. Current research is now focusing on manipulating autophagy in cancer cells in order to inhibit drug resistance and improve patient outcome. In this review, we will discuss the dual role of autophagy in melanoma and its implications in resistance to the therapies currently available for the treatment of melanoma. We will also discuss recent developments and strategies to inhibit autophagy activity in tumor cells in pre-clinical and clinical models and the importance of this as a means of re-sensitizing tumors to current therapies.

## Autophagy

**Figure 1.**
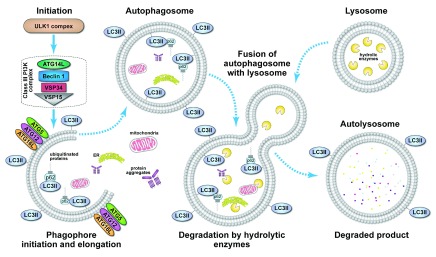
Process of autophagy. Initiation of the autophagy process is mediated by the ULK1 complex in response to various cellular signals. Formation of the phagophore also requires class III phosphoinositide 3-kinase (PI3K) complex, which is composed of VPS34 (vacuolar protein sorting 34) PI3K, ATG14L, VPS15, and beclin 1. The ATG5-ATG12-ATG16 complex and LC3II promote the elongation of the phagophore and are required for the formation of the autophagosome. p62 bound to ubiquitinated proteins targeted for degradation binds to lipidated microtubule-associated protein light chain 3 (LC3II) during the formation of the autophagosome as intracellular materials are engulfed into the forming autophagosome. Subsequently, the autophagosome fuses with a lysosome, which delivers hydrolytic enzymes for the degradation of the engulfed intracellular material.

Autophagy can be activated by various stimuli such as nutrient depletion, hypoxia, infection, stress, and aberrant cell growth during cancer. The regulation and molecular circuitry of autophagy is quite complex and involves various conserved autophagy genes. The initial step of autophagy consists of the synthesis of the phagophore, which requires the ULK1 complex and the formation of the class III phosphoinositide 3-kinase (PI3-K) complex. This is followed by the elongation of the phagophore, which requires the ATG5-ATG12-ATG16 complex and lipidated microtubule-associated protein light chain 3 (LC3) − LC3II. The growing phagophore engulfs cytoplasmic material targeted for degradation as it progressively forms a complete autophagosome. The last step of autophagy involves the fusion of the autophagosome with the lysosome and the subsequent degradation of the vesicle’s content by lysosomal hydrolases ^12^. The main stages of the autophagy process are summarized in Figure 1.

Autophagy is a dynamic process that involves numerous steps; therefore, measuring autophagy proteins and structures at any stage of the process might not be indicative of the overall changes in autophagy activity, so a measurement of flux through the entire autophagy pathway is recommended. Autophagy flux is described as the entire process of autophagy from the formation of the autophagosome to the formation of the autolysosome and subsequent degradation of the cargo and release of the degraded products into the cytosol ^13^. Autophagy flux can be measured by the use of inhibitors that block the fusion of the autophagosome with the lysosome or by increasing lysosomal pH with a lysosomal proton pump inhibitor such as bafilomycin, thus allowing the measurement of accumulated LC3II ^14^. Nascent LC3 is converted to LC3I by ATG4; LC3I in turn becomes conjugated with PE (phosphatidylethanolamine) to form LC3II. While LC3I remains in the cytoplasm, LC3II binds to both the outer and the inner membranes of the autophagosome; therefore, it is considered a marker of the autophagosome. It is important to note, however, that measurement of LC3II should be complemented with other methods such as transmission electron microscopy or fluorescence microscopy using the mCherry-enhanced green fluorescent protein (eGFP)-LC3 autophagy reporter system in order to have an accurate assessment of autophagy flux. The mCherry-eGFP-LC3B autophagy reporter system has been used in both fluorescence microscopy and the quantification of autophagy flux by flow cytometry ^15^. This fluorescent reporter is based on the principle that the GFP tag is acid sensitive, while the mCherry tag is acid insensitive. In an unfused autophagosome, both tags are expressed, such that the cell glows yellow. When there is increased autophagy, such that the autophagosome fuses with the acidic autolysosome, or endosome, GFP is degraded, and mCherry is the stronger signal, such that the cell now glows red. This allows for a quantitative, dynamic assessment of autophagy in living cells. The ability to measure this complex process effectively using various methods has led to a greater understanding of autophagy in the spectrum of cancer.

## Dual role of autophagy in melanoma

Autophagy is essential for survival and development; aside from maintaining homeostasis, this catabolic process protects organisms from various types of conditions including neurodegenerative diseases, heart disease, liver disease, and aging ^16– 18^. In the context of cancer, affecting the genes involved in autophagy, such as *ATG5* or *ATG7* and the gene *Beclin 1*, has been shown to result in tumor promotion. For example, deletion of *ATG5* or *ATG7* in the mouse induces spontaneous benign tumor formation in the liver and concomitant deletion of *p62* reduces tumor size, implying that accumulation of p62 due to deficient autophagy contributes to tumor progression ^17^. Heterozygous disruption of the *Beclin 1* gene has also been shown to cause spontaneous tumor formation and accelerate hepatitis B virus-induced neoplasia ^18^. *Beclin 1* is the mammalian orthologue of yeast autophagy-related gene *ATG6*; it is an essential component of the class III PI3K, which is required for autophagy induction. *Beclin 1* has also been found to be monoallelically deleted in human breast and ovarian cancer ^19, 20^. Mathew and colleagues investigated how the loss of *Beclin 1* contributes to tumorigenesis; they found that monoallelic loss of *Beclin 1* induces DNA damage and chromosome instability, which in turn can promote tumor initiation ^21^. Taken together, these studies show that autophagy prevents tumor initiation by protecting cells from metabolic stress that can lead to an imbalance in cellular homeostasis. However, as tumors progress, autophagy takes on a different role. Studies in various types of cancers such as colorectal cancer, glioblastoma, and esophageal cancer have shown that established tumor cells use autophagy to meet the high metabolic demands of cancer cells that are rapidly proliferating ^22– 24^.

Liu and colleagues identified the BH3-only protein Noxa as a driver of melanoma development and progression; upregulation of Noxa by MEK/ERK oncogenic activation promotes an increase in autophagy through the transcription factor cAMP responsive element binding protein (CREB). In this study, Noxa was found to be necessary for MEK/ERK-driven autophagy; moreover, Noxa was shown to induce constitutive activation of autophagy that delayed the apoptosis of human melanoma cells under nutrient-deficient conditions ^25^. Under stressful conditions, melanoma cells have been shown to upregulate autophagy activity as a protective mechanism; Marino and colleagues found that melanoma cells cultured under acidic conditions are able to survive through the upregulation of autophagy activity. Inhibition of autophagy by ATG5 knockdown decreased melanoma cell survival under acidic conditions ^26^. This highlights the fact that melanoma cells can use autophagy as an adaptive mechanism to survive and progress under the stressful conditions of the tumor microenvironment.

Aggressive tumor cells with high autophagy levels are also prone to developing drug resistance by using autophagy to escape drug-induced stresses. For instance, in esophageal cancer, inhibition of Beclin 1 and ATG7 greatly increases the effects of the chemotherapeutic agent 5-FCU ^24^. In the particular case of melanoma, inhibitors designed to target mutant BRAF fail to produce long-term effects in the clinic owing to the emergence of resistance. One recent study investigated mechanisms of drug resistance in therapy-induced autophagy; Ma and colleagues investigated resistance pathways adopted by the autophagy machinery following treatment with PLX4720, a BRAF ^V600E^ inhibitor ^27^. They found that BRAF inhibition upregulated autophagy in patients with BRAF-mutant melanoma; these patients were less sensitive to BRAF inhibition and had poorer clinical outcome. Further, in melanoma cells, both BRAF inhibition and combinatorial BRAF/MEK inhibition induced cytoprotective autophagy due to the activation of the endoplasmic reticulum (ER) stress response triggered by mutant BRAF binding to GRP78, a chaperone required for ER integrity ^27^. Overall, the combination of BRAF and autophagy inhibition was found to be efficient in inducing tumor regression in the PLX4720-resistant tumor xenografts. As mentioned earlier, another mechanism of resistance is the reactivation of the MAPK pathway through MEK; a recent study explored the effects of MEK and autophagy inhibition in BRAF-inhibitor-resistant melanomas. The authors found that inhibiting autophagy in vemurafenib-resistant melanomas either by ATG5 knockdown or pharmaceutically was not sufficient in restoring sensitivity to vemurafenib treatment; however, combination of autophagy inhibition and MEK inhibition increased melanoma cell death ^28^. Based on the aforementioned data, in recent years, many efforts have been made to unravel the effects of autophagy in melanoma tumor progression; however, there is still a need for the development of more melanoma models where tumor-specific autophagy inhibition is tested in the context of activated oncogenes, tumor suppressor gene loss, and modulated signaling pathways that drive melanoma growth and survival. This would allow the development of therapeutic strategies that most effectively halt the survival and progression of aggressive melanoma. Table 1 summarizes studies based on *in vitro* and *in vivo* models using autophagy inhibition or combinatorial therapies in melanoma.

**Table 1.** Strategies to inhibit autophagy and increase drug-induced death in melanoma.

Model	Therapeutic combinations	Outcome	Reference
*In vitro*	siATG5/MEK inhibitor (U0126)	Increased cell death (BRAF- inhibitor-resistant lines)	28
Tg ^Tyr-cre/ERT2/+^, LSL-BRAF ^V600E/+^, Pten ^FLOX/FLOXAtg7+/+^, or Atg7 ^FLOX/FLOX^	ATG7 deficiency/dabrafenib	Decrease in tumor growth/ increase in senescence	29
Three-dimensional culture, tet- inducible shATG5	ATG5 deficiency/temozolomide	Increased cell death	30

## Inhibiting autophagy to control melanoma tumor progression

In recent years, autophagy modulation through genetic knockdown of major autophagy genes or through the use of pharmacological inhibitors has been widely used with the goal of sensitizing tumor cells to metabolic stress. Using a genetically engineered mouse model (GEMM), Mehnert and colleagues showed that tumor-specific deletion of *ATG7* in *BRAF ^V600E^* and *Pten*-deficient melanomas suppressed tumor growth and extended survival of mice ^29^. In this model, combination of ATG7 deficiency and dabrafenib treatment significantly decreased melanoma tumor growth and induced senescence ^29^. These findings highlight the dependency of tumor cells on autophagy to survive and progress. Another study correlated autophagy activity with patient outcome in the clinic and proposed that autophagy is a potential prognostic factor for predicting melanoma cell invasiveness and patient outcome ^30^. In this study, autophagy was measured in tumor biopsies obtained from metastatic melanoma patients enrolled in a phase II trial of temozolomide and sorafenib. Overall, patients whose tumors displayed high autophagy levels had a poorer clinical outcome compared to patients with low autophagy activity in their tumors. This positive correlation between autophagy activity and tumor aggressiveness was recapitulated in a tumor xenograft model where aggressive melanoma tumor xenografts displayed a higher autophagy flux compared to the indolent ones. Furthermore, autophagy inhibition by genetic knockdown of *ATG5* or hydroxychloroquine (HCQ) treatment increased temozolomide-induced cell death in 3D culture ^30^. An important point to note from the latter study is that not all melanoma patients will benefit from a treatment that targets autophagy; autophagy inhibition or combinatorial treatments involving autophagy inhibition will more likely benefit those patients whose tumors have high autophagy activity. It is also important to note that therapy can increase autophagy activity in patients’ tumors, and while it is known that therapy-induced autophagy can lead to the development of drug resistance, it is crucial to further dissect mechanisms by which autophagy induces drug resistance in order to have a rationale for the development of combinatorial therapies.

## Autophagy inhibition for cancer therapy: clinical trials

In the clinic, the emergence of therapy resistance in melanoma and other cancers is a major problem. This has prompted immense efforts to develop autophagy inhibitors designed to increase chemosensitivity. In recent years, phase I/II clinical trials that involve HCQ-mediated autophagy inhibition have been undertaken in various cancers including melanoma, pancreatic cancer, and myeloma ^31– 34^. These trials evaluated maximum tolerated doses, pharmacodynamics, clinical activity, and toxicities in patients. HCQ is an anti-malarial drug that has been shown to inhibit the late stage of autophagy ^35^. A phase I clinical trial of HCQ and temozolomide involved 40 patients with advanced solid tumors, 73% of whom had metastatic melanoma; >4 months of prolonged stable disease was observed in patients with metastatic melanoma. Partial responses and stable disease were observed in 14% (3/22) and 27% (6/22) of patients with metastatic melanoma, respectively. Pharmacodynamic activity of the combined therapy *in vivo* was measured by a significant accumulation of autophagy vacuoles in peripheral blood mononuclear cells ^31^. Another phase I clinical trial in patients with advanced solid tumors and melanoma involved the combination of HCQ with the mTOR inhibitor temsirolimus. While no responses were observed, the majority of patients treated achieved stable disease. In 13 melanoma patients treated with HCQ at 1200 mg/d in combination with temsirolimus, the median progression-free survival was 3.5 months. In addition to measuring toxicity, this trial utilized FDG-PET measurements, which predicted clinical outcome and provided supporting evidence that this therapy increased metabolic stress in the tumors of patients who experienced clinical improvement ^32^. Clinical trials involving HCQ have also been performed in other cancers such as pancreatic adenocarcinoma, where HCQ produced negligible anti-tumor effects due to inconsistent autophagy inhibition ^33^. In myeloma, the combination of the proteasome inhibitor bortezomib and HCQ in 22 patients with relapsed or refractory myeloma resulted in 14% partial responses, 14% minor responses, and 45% stable disease ^34^. Similar to other trials, the combination of HCQ and bortezomib resulted in accumulation of autophagy vacuoles as a pharmacodynamic marker of autophagy modulation. The aforementioned clinical trials show that therapy-induced autophagy inhibition is achievable and that there has been considerable progress in the modulation of autophagy for cancer therapy ( Table 2). However, these studies also reveal that there is still a need for novel autophagy modulators that more consistently inhibit autophagy and induce tumor regression.

**Table 2.** Clinical trials involving hydroxychloroquine (HCQ)-mediated autophagy inhibition.

Cancer type	Therapeutic combinations	Outcome	Reference
Melanoma	Phase I temozolomide/HCQ	Accumulation of autophagy vacuoles	31
Melanoma	Phase I temsirolimus/HCQ	Metabolic stress on tumors	32
Pancreatic adenocarcinoma	Phase II HCQ	Inconsistent autophagy inhibition	33
Myeloma	Phase I bortezomib/HCQ	Increase in autophagy vacuoles	34

## Conclusions

Although it is established that autophagy has a dual role in cancer, the dynamics by which this catabolic process suppresses or promotes cancer still remain complex. Efforts to unravel molecular and cellular mechanisms that might modulate autophagy activity in cancer cells have started to emerge. The overall findings reveal that the consequence of defective autophagy in the context of cancer is fundamentally contingent upon the stage of cancer development. Deficient autophagy can induce metabolic stress, which can lead to the accumulation of protein aggregates and dysfunctional mitochondria; this disruption of cellular homeostasis can in turn induce genomic instability and promote cancer development. On the other hand, many studies have now showed that in advanced stages of cancer, autophagy can promote therapy resistance and survival of aggressive tumor cells. Hence, the challenge lies in specifically targeting autophagy in the right subpopulation of tumor cells, and in the clinic it makes scientific sense that patients whose tumors have high basal autophagy will be the ones who benefit most from autophagy inhibition. A major challenge that also requires attention is the need for autophagy inhibitors that consistently inhibit autophagy and convey potent anti-tumor activity. In the particular case of melanoma, pre-clinical models and clinical trials have indeed demonstrated the potential of autophagy inhibition for therapy; however, more studies are needed to dissect the mechanisms that regulate autophagy in aggressive melanoma cells for the development of therapeutic strategies that will benefit patient outcome.

## Abbreviations

CTLA-4, cytotoxic T-lymphocyte-associated protein 4; ER, endoplasmic reticulum; eGFP, enhanced green fluorescent protein; HCQ, hydroxychloroquine; IGFR, insulin-like growth factor receptor; LC3, microtubule-associated protein light chain 3; MAPK, mitogen-activated protein kinase; PI3-K, phosphoinositide-3-kinase; PD-1, programmed cell death protein 1.
